# Phage Display: Selecting Straws Instead of a Needle from a Haystack

**DOI:** 10.3390/molecules16010790

**Published:** 2011-01-19

**Authors:** Miha Vodnik, Urska Zager, Borut Strukelj, Mojca Lunder

**Affiliations:** 1 Department of Pharmaceutical Biology, Faculty of Pharmacy, Aškerčeva 7, Ljubljana, Slovenia; 2 Department of Rheumatology, University Medical Centre Ljubljana, Vodnikova 62, Ljubljana, Slovenia; 3 Department of Biotechnology, Jozef Stefan Institute, Jamova 39, Slovenia

**Keywords:** phage display, target-unrelated peptides, decoy

## Abstract

An increasing number of peptides with specific binding affinity to various protein and even non-protein targets are being discovered from phage display libraries. The power of this method lies in its ability to efficiently and rapidly identify ligands with a desired target property from a large population of phage clones displaying diverse surface peptides. However, the search for the needle in the haystack does not always end successfully. False positive results may appear. Thus instead of specific binders phage with no actual affinity toward the target are recovered due to their propagation advantages or binding to other components of the screening system, such as the solid phase, capturing reagents, contaminants in the target sample or blocking agents, rather than the target. Biopanning experiments on different targets performed in our laboratory revealed some previously identified and many new target-unrelated peptide sequences, which have already been frequently described and published, but not yet recognized as target-unrelated. Distinguishing true binders from false positives is an important step toward phage display selections of greater integrity. This article thoroughly reviews and discusses already identified and new target-unrelated peptides and suggests strategies to avoid their isolation.

## 1. Introduction

Phage display, a powerful tool for discovering ligands for various targets, was first described in 1985. Since then, the technique has evolved and has become widely used in basic and applied biosciences to study molecular biology mechanisms involving protein-protein [1] or protein-nonprotein interactions [2]. A phage display library is a complex mixture of phage clones displaying random peptides, foreign proteins or protein domains genetically fused with phage surface proteins [3]. Filamentous phage with displayed peptides fused to the N-terminus of capsid proteins p3 or p8 are typically used; however, several alternative phage display formats aiming to avoid the limitations of the conventional display system are also available (for a detailed review see [4]). Commercially available phage display libraries based on filamentous phage M13 (Ph.D.^TM^-7, Ph.D.^TM^-12, Ph.D.^TM^-C7C; New England Biolabs) or spherical T7 phage (T7Select^®^, Merck) are most commonly used. Researchers sometimes construct their own phage libraries, especially when specific library characteristics are required. Large phage populations can be screened in a process called biopanning to yield ligands that exhibit the desired target behavior. Phage clones from the library are incubated with the immobilized target molecule. Nonbinding virions are removed by multiple washing steps and phage bound to the target are recovered by different elution strategies. The most common approach is nonspecific elution (acidic buffers, DTT, high ionic strength or ultrasound) which tends to weaken the interaction between the virion and the target [3]. Elution of background-bound phage can be avoided with competitive elution when ligands of the target are known. Phage can also be eluted competitively but nonspecifically by using free target or adding bacterial host [3]. A typical screening procedure involves several rounds of biopanning, until phage pool is enriched in specific binding phage.

Even with many successful selections and discovery of strong ligands for various targets described in the literature, ambiguous results often appear. Instead of specific binders, phage with no actual affinity toward the target may be recovered. A typical phage display peptide library contains 10^9^-10^10^ different clones. A vast majority of clones do not bind to the target molecule or to any other component of the screening system and are removed during washing steps. A small proportion of phage bind specifically to the screening target, and only a few of them are capable of high-affinity binding. These represent the expected result of biopanning. However, phage binding to other components of the screening system, such as contaminants in the target sample, solid phase (plastic plates, beads), capturing reagents (streptavidin, protein A/G, biotin, secondary antibody), substances used for blocking the solid surface (bovine serum albumin, milk) or any other constituent, may predominate during rounds of biopanning. Peptides binding to other components of the screening system rather than the target can also be classified as selection-related target-unrelated peptides [5]. Many such peptides have already been identified. Menendez and Scott recently reviewed a collection of target-unrelated peptides recovered in the screening of phage-displayed random peptide libraries with antibodies [6]. 

Another type of target-unrelated peptides are propagation-related [5,7]. Here selection is driven by faster propagation rate of some phage clones. Recovery of such clones is independent of their affinity because the advantage in replication enables them to prevail in the phage pool. Faster propagation of certain phage clones may result from a mutation in the phage genome, which influences the ability of the virus to infect host bacteria or accelerates the process of phage particle assembly. It may also be an intrinsic property of the displayed peptide itself, without any causative mutation [5,8].

Target-unrelated peptides are false positive results regardless of their origin, and have to be recognized and distinguished from true positive clones. Detailed review of the literature has revealed numerous target-unrelated peptides. For most of them the cause of their predominance has been determined, either by revealing the decoy in the screening system or by demonstrating their propagation advantages. They can therefore be included in either the selection-related or propagation-related groups. However, this is not the case for all target-unrelated peptides. Biopanning experiments on different target molecules performed in our laboratories revealed some previously identified and many new target-unrelated peptide sequences, which have already been frequently described and published but had not yet been recognized as target-unrelated. Moreover, some of these peptides have been mistaken for true target binders. This article thoroughly reviews and discusses already acknowledged and new target-unrelated peptides.

## 2. Selection-Related Target-Unrelated Peptides

One of the most important factors influencing selection of unrelated peptides is target immobilization. Adsorption to plastic polystyrene microtiter wells or Petri dishes [9] is the most common approach, but many other capturing procedures exist. Target molecules can be biotinylated and affinity-captured on streptavidin coated plates or beads [3,10]. A common way to immobilize antibodies is capturing the Fc region to protein G, protein A [11] or secondary antibodies adsorbed to the solid surface [6]. His-tagged proteins can be immobilized to metal-ion chelated microtiter plates or beads [12,13]. Epoxy beads have also been used [14]. A self-assembled monolayer on gold was developed for the attachment of small organic molecules [15]. To prevent interactions of library phage with the solid surface or coupling reagent, the immobilized surface is often additionally covered with a blocking agent, such as bovine serum albumin, powdered skim milk, gelatin or casein. Each of these components may divert affinity selection away from the target molecule.

### 2.1. Plastic Binders

Proteins (peptides) and other non-protein targets adsorb nonspecifically and noncovalently to polystyrene surfaces via hydrogen bonds and nonpolar interactions. Adsorbed proteins are partially denatured [16], which results in nonspecific hydrophobic interactions of aromatic amino acid residues with aromatic polystyrene phenyl moieties [9,17]. Virtually all proteins bind to plastic to some extent. Likewise, phage clones displaying certain peptides show greater affinity for plastic surfaces [6]. Such clones stick to the plastic surface, withstand washing steps and are subsequently amplified. Covering unoccupied polystyrene surfaces with a blocking agent does not necessarily prevent a predominance of plastic binders. 

A common feature of plastic binding peptides is a high abundance of aromatic amino acid residues [6,9], which are crucial for hydrophobic interactions. Various similar consensus motifs have been reported, demonstrating that distribution of aromatic residues (most notably W) is not random. Two W residues separated by one, two or three random amino acids frequently occur [9,18]. Clones displaying peptides with typical motifs emerged from selections on various targets (Table 1) performed by various groups, including our own. In some selections aiming, for example, to discover peptide affinity tags, the plastic surface was the actual target [9,19,20,21]. Some groups have recognized plastic binders as such; however, some claim that their peptides bind specifically to the target. Thus, peptides showing strong resemblance to motif FHXXW peptides, FHQNWPS peptide (selected in biopanning on bovine serum albumin [22]) and PHWTWVL peptide (selected on chromatin high mobility group protein 1 [23]) could well be plastic binders. With no convincing evidence of affinity, selection was indeed most likely diverted from the primary target. 

However, enrichment of aromatic amino acid residues must not automatically be considered as plastic binding. Peptides that are not enriched in aromatic residues may also bind to plastic, whereas some peptides containing many hydrophobic residues bind specifically to their corresponding targets. Furthermore, binding to plastic does not necessarily exclude the ability to bind to certain targets. Confirmed plastic binder FHENWPS interacts with the CD23 surface receptor expressed by variety of cells [24]. FHENWPS was also isolated by selection on cellulose and carbon black, a component of ink. Specific binding was confirmed on carbon black, but not on cellulose. In the case of cellulose it is possible that FHENWPS was selected as a result of nonspecific binding to the background, whereas in the case of highly hydrophobic carbon black, binding most likely occurred through nonspecific hydrophobic interactions, rather than specific binding to certain chemical moieties. This notion is further supported by an alanine scan of the selected peptides. Replacement of W, H and F by A significantly reduced carbon binding, indicating the importance of the aromatic residues [25]. The FHENWPS peptide has also been proposed to interact with the glycolipid globotriaosylceramide, which is important for internalization of the Shiga toxins of Escherichia coli [15]. The peptides FYSHSFHENWPS and FHENWPSGGGSA selected from a 12-mer library using a globotriaosylceramide mimic as the target, were able to inhibit Shiga toxin binding. Considering the affinity of FHENWPS for hydrophobic surfaces, these peptides probably bind to the lipid and not the carbohydrate moiety of globotriaosylceramide.

### 2.2. Albumin Binders

Albumin is known to weakly bind various molecules, including hormones [26], acidic and lipophilic drugs [27], fatty acids [28], bilirubin [29] and metal ions [30]. Albumin isolated from bovine serum is commonly used as a blocking agent. However, when biopanning on targets without strong hotspots for protein interactions, albumin must be considered as a possible decoy due to various binding sites on its surface. 

Albumin samples from humans, rabbits and rats were used as targets for phage display selection with the aim of developing peptide linkers capable of extending plasma half-life of therapeutics through albumin binding [31]. Phage clones selected on albumin from various species expressed highly similar peptides with the core consensus sequence DICLPRWGCLW. Cross-reactivity of peptides with albumins from other species including bovine serum albumin was observed probably due to the more than 70% sequence similarity of these albumins. No cross-reactivity was observed with structurally different ovalbumin. The synthetic peptide Ac-RLIEDICLPRWGCLWEDD-NH_2_ actually had a longer half-life after intravenous injection than a random peptide of the same length, which further confirmed binding to albumin. Additional phage-displayed peptides (Table 1) with convincingly confirmed affinity to human serum albumin were selected by Sato *et al.* [32]. In selection described by Desjobert *et al.* [22], bovine serum albumin was used as target. No common consensus motif emerged and none of the phage clones predominated. Peptide FHQNWPS, which has been discussed above as a possible plastic binder, and peptide HWGMWSY appeared in selections on several other targets and is further discussed in a later section of this review. Other peptides (Table 1) showed no resemblance to the consensus sequence described by Dennis [31] or to the peptides selected by Sato [32].

### 2.3. Streptavidin and Biotin Binders

Streptavidin, a tetrameric protein with four high affinity biotin binding sites (K_D_ ~10^-14^), is often used to capture biotinylated targets on solid surfaces such as polystyrene microtiter wells [33], magnetic beads [34] or gold surfaces [35]. This capturing approach is also widely used in biopanning experiments. However, streptavidin and biotin are additional components of the screening system that may divert selection from the target.

Streptavidin binding peptides have been well characterized, and an overview is presented in Table 1. The most recognizable tripeptide motif, HPQ, binds to the same binding site as biotin [36]. Constrained and linear peptides of different lengths containing HPQ at different locations within the amino acid sequence were selected from phage display and other libraries [33,34,36,37,38,39]. Other amino acids in these sequences do not seem to play a significant role in binding, because little similarity can be observed in residues flanking the HPQ motif. However, some amino acid residues are preferential at the C-terminal side of the HPQ motif. The most frequent residues are F (34%) and G (25%), followed by N (15%) and V (10%) [6]. 

**Table 1:** Peptides binding to different components of screening systems.

Intended target and reference		Consensus binding motif/peptide	Confirmed or suspected decoy
Polystyrene [9]			
Peptide GREPRVATVTRILRQ [59]		WXXW	Plastic
Ghrelin (our work)			
Streptavidin [18]			
Chromatin high mobility group protein 1 [23]		WHXW	Plastic
Ghrelin (our work)			
Peptide GREPRVATVTRILRQ [59]		WXXWXXXW	Plastic
Polystyrene [19]		FKFWLYEHVIRG	
Polystyrene [20]		RAFIASRRIKRP	Plastic
Polystyrene [21]		FHWTWYW	
Chromatin high mobility group protein 1 [23]			
Penicillin binding proteins [60]		WHW	Plastic (suspected)
Microcystin-LR [61]			
Ghrelin (our work)			
Gb3 mimic [15]			
HIV I integrase [22]			
sCD23 protein [24]			
Cellulose, carbon black [25]		FHENWPS	Plastic (suspected)
Monoclonal antibody DJ2.8 [62]			
Hydroxyethyl- and acetaldehyde-modified bovine serum albumin [63]			
OmpF porin [64]			
Human, rat, rabbit serum albumin [31]		DICLPRWGCLW	Albumin
		VAWCTIFLCLDV	
Human serum albumin [32]		FWFCDRIAWYPOHLCEFL	Albumin
		RNMCKFSWIRSPAFCARA	
		NPRLYE	
		TTYSRFP	
		AEPVAML	
		VPTGYKP	
		ASSRTPS	
Bovine serum albumin [22]		HFWNRPL	
		SPYHTQP	
		LPPNPTN	
		HAWNYIF	
		NSHAIYP	
		SLLSSPQ	
		FHQNWPS	Plastic (suspected)
		HWGMWSY	Plastic/propagation-related (suspected)
	[33], [34], [37]^1^, [39]^2^	HPQ	
	[18]	EPDW(F/Y)	
Streptavidin	[39]^2^	DVEAW(L/I)	Streptavidin
	[40]	GD(F/W)XF	
		PWXWL	
Biotin	[41]	WXPPF(K/R)	Biotin
		HTHHHT	
		HHHHPT	
Ni^2+^, Zn^2+^, Cu^2+^ [12]		HHHHHT	
		HHHHTN	
		HHTHSL	
Co^2+^ [13]		KSLSRHDHIHHH	Bivalent metal ions
Cu^2+^ [42]		GRVHHHSLDV	
		SWKHHAHWDV	
Zn^2+^ [42]		SHTHALPLDF	
		GRVHHHSLDV	
Zn^2+^ [43]		RSDRGKAHPSRRS	
		RSVHASHHHIMRS	
Protein A [11]		WT(I/L)XXHR	Protein A
Human IgG auto-anti IgE [49]		RPSP	SRPSP Cε2-Cε3 interdomain of human immunoglobulin ε-chain
Goat anti-human IgM [50]		GLTFQ	Human µ-chain
Goat anti-mouse IgG [51]		RT(I/L)(S/T)KP	Fc region of mouse γ-chain
Fc fragment of human IgG [52]		SS(I/L)	
Fc fragment of human IgG [55]		GELVW	
Fc fragment of a humanized monoclonal IgG1 [65]		SS(I/L)	
		EPIHRSTLTALL	Fc region of IgG
Murine IgM monoclonal antibodies [66]		WI(S/P)(S/Q)XDW	
Synthetic peptide [67]		GQVLQGAIKG	
Synthetic peptide [68]		RTY	
Human anti-HIV monoclonal antibodies b12, CB-1, 4E10, polyclonal IgG from HIV-1 infected donor, mouse monoclonal antibody PAK-13 [6]		SS(I/L)	Fc region of Ig (suspected)
Bovine anti-p34 antibodies [54]			
Biotinylated anti-dsDNA antibodies [53]			
GS-1-B4 lectin [69]			
Human IgG-Fc [56]		CTGYWPKAWGLC	Non-native conformation of IgG
Monoclonal antibodies 2G12, b12, HyHEL-10 [6]			
Monoclonal antibodies F-105, 4b4, 449, GZD1E8 and bovine IgG [57]		QSYP	Bovine IgG
Human autoimmune MAb T13 [58]			

^1^ mRNA display, ^2^ ribosome display

Streptavidin binding peptides without the HPQ motif have also been identified. Peptides with the consensus sequence DVEAW(L/I) along with HPQ were selected from a ribosome display library of 15-residues-long random peptides [39]. Other streptavidin binding motifs include EPDW(F/Y) [18], GD(F/W)XF and PWXWL [40]. 

Competition assays with biotin [39] and an HPQ peptide [18] showed that these peptides also bind to the biotin binding pocket on streptavidin. Peptides binding to biotin have also been identified. Biotin, in free soluble form, is sometimes added after coupling a biotinylated target to streptavidin in order to fill remaining binding sites on streptavidin. Furthermore, biotinylation of the target molecules is often multivalent. Some biotinylated side groups are exposed to the solvent and are capable of attracting phage particles. Saggio [41] isolated peptides displaying a WXPPF(K/R) motif from a 9-mer p8 phage displayed library using a biotinylated monoclonal antibody against nicotinic acetylcholine receptor. Selected peptides did not recognize nonbiotinylated antibody but specifically reacted with other biotinylated antibodies (human IgG, goat anti-rat IgG) and biotinylated bovine thyroglobulin.

### 2.4. Bivalent Metal Ion Binders

Use of transitional metal ions (Co^2+^, Zn^2+^, Cu^2+^ and Ni^2+^) chelated on solid surfaces is a quick and efficient approach for immobilization or affinity purification of His-tagged proteins. Site directed immobilization enables proper orientation of the target, which retains its native conformation. Immobilization to Co^2+^, Zn^2+^ or Ni^2+^ surfaces has also been successfully used in phage display experiments. Studies on natural metal binding proteins have revealed that almost half of 20 naturally occurring amino acids contribute to metal binding [42]; the most notable among them is the histidine residue. Histidine forms a strong coordination bond between nitrogen in an imidazole ring (donor of electron pair) and a positively charged metal ion (acceptor). Therefore, enrichment of target-unrelated histidine-rich peptides is a frequent by-product of such selections. This enrichment has also been noted in our laboratory. However, the presence of histidine is not obligatory for binding to bivalent metal ions. In the selection of antibody fragments on different metal ions, among them Zn^2+^ and Cu^2+^ from semisynthetic combinatorial antibody libraries, some selected antibody fragments were devoid of histidine [42]. Peptides lacking histidine but possessing an affinity for Zn^2+^ also emerged among histidine-enriched peptides in screening a bacterial display library of random peptides [43]. Furthermore, histidine-enriched peptides may not necessarily be target unrelated, which was evident for peptide KSLSRHDHIHHH. This peptide was selected using Co^2+^ beads to immobilize the autoreactive antibodies present in sheep with Johne’s disease [13]. Although not determined by the authors, this peptide most likely possesses affinity for bivalent metal ions as well.

### 2.5. Protein A and Protein G Binders

Screening of large phage display peptide libraries is an effective approach for mapping epitopes of monoclonal or polyclonal antibodies. Selected peptides reflect linear [44,45] or conformational [46,47] epitopes that are recognized by antibodies. Identification of antigen epitopes and allergens is crucial for understanding the pathogenesis of autoimmune diseases and allergies. Screening of antibodies recognizing microbial antigens enables identification of short peptides mimicking epitopes (mimotopes). Such peptides can trigger a similar immune response as corresponding antigens and are therefore candidates for new vaccines [48]. Direct adsorption of antibodies directly to a plastic surface may result in improper orientation or denaturation and hence decreased antigen binding capacity. Therefore staphylococcal protein A or streptococcal protein G, which interact with the heavy chain of the Fc portion of IgG, are often used for immobilization. Protein A and G enable specific and effective antibody capture, but may also bind phage clones resulting in unwanted selection of peptides unrelated to the target. Thus far, only peptides binding to protein A have been identified [11]. For selection on anti-actin polyclonal antiserum, a protein A column was used for purification of the antibody-phage complex. A large proportion of phage clones was found to interact with protein A instead of the screened antiserum. Peptides binding to protein A contained the consensus motif WT(I/L)XXHR. Whether the peptide binds to the same spot as the Fc region of antibodies was not determined.

### 2.6. Secondary Antibody Binders

An alternative method for immobilizing screening antibodies is affinity capture to secondary antibodies. This approach is less convenient because phage particles are likely to bind to the secondary antibody resulting in selection of target-unrelated peptides. Peptides recognizing epitopes of secondary antibodies have been intentionally selected from phage libraries by several groups. All peptides corresponded to the amino acid sequence found in Fc regions of matching primary antibodies (Table 1) [49,50,51].

### 2.7. Peptides Binding to Immunoglobulin Fc Region

Immunoglobulins are massive proteins, with molecular weight around 150 kDa (IgG), but the antigen binding site is limited to only a small proportion of variable region. Phage clones with affinity to non-binding areas of the variable region or to the constant region may emerge or predominate in the phage pool during screening. This outcome is favored when an antibody recognizes a discontinuous epitope [6]. 

In several screenings on human or animal antibodies or antibody fragments, peptides with motif SS(I/L) appeared (Table 1). Studies have revealed that peptides containing SS(I/L) bind to the Fc region of antibodies. A systematic search for Fc binding peptides by specific elution with protein A yielded peptides with the common motif F(A/G)RLV**SSI**RY, which competed for the protein A binding site [52]. This site on the Fc fragment seems to be preferential for interactions because the same motif emerged in experiments where nonspecific elution was applied [52,53,54]. 

Other motifs with affinity towards the protein A binding site on the Fc region also appeared. These motifs include TWKTSRISIF, which was selected by specific and nonspecific elution [52], and GELVW (DCAWHLGELVWCT), which was selected by nonspecific elution; both motifs are located on the human Fc region [55]. The peptide motif GLT(D/R)(W/Y) with unknown specificity was also isolated and, according to the authors, probably binds to the constant region of the human HIV-1-neutralizing monoclonal antibody 2F5 [6]. Peptides selected on the Fc region of human IgG show some cross-reactivity with immunoglobulins of other species. The peptide FGRLV**SSI**RY interacts strongly with human and porcine, but weakly with murine, sheep and rabbit Fc regions of IgG. The peptide TWKTSRISIF interacts moderately with the porcine Fc region of IgG [52].

### 2.8. Peptides Binding to Impurities Found in Antibody Preparations

Antibody preparations may contain impurities, such as partially denatured antibodies, components of cell growth media and others to which phage clones may bind during selection. Peptides such as CTGYWPKAWGLC were shown to bind with high affinity to the non-native conformation of IgG, an impurity that appeared as a consequence of acidic elution from protein A during isolation [56]. Interestingly, an acidic conformer retained its antigen binding activity as well as Fc receptor binding. A consensus peptide sequence, QSYP, has appeared as an artifact during the mapping of different monoclonal antibodies using phage display in different laboratories [6,57,58]. A decoy was confirmed to be bovine IgG derived from the fetal bovine serum present in the hybridoma growth media [57]. 

## 3. Propagation-Related Target-Unrelated Peptides

Another group of phage clones unrelated to the target may predominate in the phage pool during screening. These clones emerge due to their advantage in propagation that allows them to outgrow other clones in the library. Faster propagation can be a result of increased infectivity or production of phage particles. During the amplification step after each round of biopanning such phage clones achieve higher titer [7]. Within a few rounds of biopanning, these clones may predominate although they are unrelated to the target or any other component of the screening system. Faster propagation may be a result of the displayed peptide itself (intrinsic) or a mutation in the phage genome (extrinsic) [5]. Surface-displayed peptides diminish phage infectivity to some extent. Certain phage clones replicate slower because displayed sequences impede the phage assembly process to a greater extent. It is impossible to display some peptide sequences on the surface of phage, because they are not compatible with the phage replication process. On the other hand, some sequences allow a relatively faster propagation rate, and such clones are therefore more likely to be isolated. In M13-based libraries, P is an example of amino an acid residue that is overrepresented, whereas C is under-represented [8]. This phenomenon is known as sequence bias. Peptides with biased amino acid composition may nonetheless specifically bind to a certain target. Their selection is due to different rates of propagation, which does not necessarily exclude specific selection in the presence of an appropriate target.

Phage clones that propagate faster due to mutation in the phage genome, are unlikely to specifically bind to the target. A few have already been identified and are listed in Table 2. HAIYPRH was selected from a 7-mer phage display library Ph.D.-7^TM^ on various targets in different laboratories, including our own. The phage clone displaying the peptide HAIYPRH in the Ph.D.-7^TM^ library amplifies at a dramatically faster rate than other clones due to a G→A mutation in the Shine-Dalgarno sequence of p2, a protein involved in the replication process [7]. This mutation increases expression of p2, which results in faster replication of the phage genome and speeds up the entire phage generation process [70]. Any peptide would be able to predominate, if displayed on phage clone with this mutation. The presence of the HAIYPRH is merely a coincidence. Although a mutated clone is present in a commonly used commercial phage display library, it is not consistently enriched. Selection of rapidly propagating target-unrelated peptides is favored when only low/moderate affinity ligands for a specific target are present in a phage display library. 

Surprisingly, specific affinity of HAIYPRH for a certain targets [23,71,72,73] has been demonstrated. Lee *et al.* [71] identified HAIYPRH as a specific ligand for the human transferrin receptor. The synthetic peptide activated endocytosis via the transferrin receptor and is a promising candidate carrier for the intracellular delivery of therapeutics [74,75]. 

**Table 2:** Propagation-related target-unrelated peptides.

Selected peptide	Intended target	Reference
RGGRCLLCCLCLWWA		
AVAGGRSVVDARVAR	Mice harboring MDA-MB-435eb.1 xenograft tumors	[5]
RTEVPVLSFTSPLTG		
	Zn^2+^	[7]
	Chromatin high mobility group protein 1	[23]
	Transferrin receptor	[71]
	A549 bronchial epithelial cell line	[72]
	Umbilical veins	[73]
	Monoclonal antibody against hepatitis E virus	[77]
	Isotactic polymethyl methacrylate film	[78]
	Clostridium difficile toxin A	[79]
	Control selection (no target)	[80]
HAIYPRH	Small molecular ink	[81]
	Polyclonal serum against Cucumber mosaic virus	[82]
	Crystalline cellulose	[83]
	Factor interacting with poly(A) polymerase (Fip1)	[84]
	Printing paper	[85]
	Murine muscle	[86]
	Mouse heart and skeletal muscle	[87]
	Saccule and utricle cultures isolated from C57B1/6 mice	[88]
	Angiostatin	[89]
	HepG2 cells	[90]

Fd-tet vector-derived phage display libraries are a less likely source of propagation-related clones. Here the minus strand origin of replication is disrupted by a large tetracycline resistance cassette and as a result, formation of the minus strand and consequently the replicative form is very slow. The propagation rate of fd-tet phage is severely limited, determined by the synthesis rate of minus strand ssDNA. Simple mutations are not enough to restore the propagation rate. Deletion of the tetracycline cassette could lead to restored propagation, but bacteria infected with such clones would not survive in media containing tetracycline [76]. Nevertheless, rapidly propagating clones have recently been identified in fd-tet based libraries as well [5]. *In vivo* selection using a random 15-mer peptide library on p3 in tumor-bearing mice enriched two phage clones displaying the peptides RGGRCLLCCLCLWWA and AVAGGRSVVDARVAR with no particular affinity, and these clones formed larger and less turbid plaques. Genome sequencing revealed that they were chimeric clones resulting from a very rare combination of recombination events involving a wild-type fd phage and another (not wild-type) clone in the library. These clones had a reversed sequence for tetracycline resistance in combination with a restored minus strand ori sequence. Therefore, they were able to propagate at a much faster rate in medium containing tetracycline because tetracycline resistance was not affected. To prevent outgrowth of faster propagating clones, serial amplifications of a library should be avoided. When subtractive selection is employed in a biopanning procedure with an aim to reduce binding to background, amplification after prepanning should also be avoided, provided that the number of phage is sufficient. 

## 4. Frequently Isolated Peptides/Motifs of Unknown Specificity

### 4.1. Peptide HWGMWSY

The phage clone displaying HWGMWSY has been isolated by several groups, including our own, from the M13 phage display library Ph.D.-7^TM^ using completely different protein and nonprotein targets (Table 3). Specific binding to all of these targets is highly improbable, although for some of them, binding was more or less convincingly confirmed [23,91,92]. However, interaction of short peptides with different proteins is possible because they usually recognize a very small interaction surface on a particular protein. This surface is not necessarily linear, but can also be a topological discontinuous epitope. If this is the case for the peptide HWGMWSY, a conserved hot-spot region present on different apparently unrelated proteins should exist. 

Several more plausible explanations could account for the emergence of the HWGMWSY-displaying clone in various selections. First, HWGMWSY could interact with a component of the screening system present in all described selections. This component may actually be bovine serum albumin, a common blocking agent for phage display, as proposed by Desjobert *et al.* [22]. However, as already mentioned, this motif shares no similarity with other albumin-binding peptides [31,32]. Nevertheless, the existence of distinct binding sites could explain this dissimilarity. Another common component of all the selections with the exception of one [91] is polystyrene. Because the peptide HWGMWSY contains the confirmed plastic binding motif WXXW (Table 1) and plenty of hydrophobic residues, it is much more likely to bind to the plastic surface than the albumin. Furthermore, other confirmed plastic binders and peptides that have abundant aromatic residues have been co-selected in some selections mentioned in Table 3, such as FHQNWPS [22], HSWLWWP, WHWWPxL, NWGMWSY and others [23] (amino acid marked with x was not determined in the article). HWGMWSY and structurally related co-selected clones WHWRLPS, WHWWPGM, WHFSWWP and HWWTWA were tested in our laboratory and indeed showed increased background binding. Another possible explanation for HWGMWSY displaying clone prevalence is propagation advantage. A mutation or simply an intrinsic property of the displayed peptide resulting in a faster propagation rate would also explain why the same sequence has not been isolated from any other phage display library. Furthermore HWGMWSY is always selected in one or more copies but never as a part of a motif matching with other peptides, with the exception of peptide NWGMWSY found by Dintilhac [23]. Confirmed propagation related peptide HAIYPRH was also co-selected twice in the above mentioned experiments [23,77].

### 4.2. Peptide Motif K(L/V)WX(I/L/V)P

The K(L/V)WX(I/L/V)P motif came to our attention during the characterization of anti-β2-glycoprotein I (β2GPI) antibody paratopes using the Ph.D.-7^TM^ library. Sixty percent of sequences, selected by three rounds of biopanning against β2GPI using specific elution with various anti-β2-glycoprotein I antibodies, corresponded to this motif. All selected phagotopes exhibited substantial affinity towards β2GPI, whereas no binding to bovine serum albumin was observed. Surprisingly, similar sequences were obtained, in an unrelated study performed in our laboratory, involving identification of lipoprotein lipase, pancreatic phospholipase [93] and beta-ketoacyl-ACP reductase binders from the Ph.D.-12^TM^ library (Table 3). Moderate affinity of phage displaying these peptides towards lipoprotein lipase was determined, but not towards pancreatic phospholipase. 

A stringent review of literature revealed that several other groups studying different targets also obtained similar or identical sequences (Table 3). A cyclized form of KLWTIPQ, isolated by Mizuguchi *et al*., was shown to antagonize function of IL-6 through specific binding to the cytokine [94]. The heptapeptides analogous to the discussed motif also displayed significant binding to mungbean’s heat shock proteins [95] and to the *E. coli* FtsA protein [96]. The respective motif was further selected on the N-terminal domain of human topoisomerase I [97], chromatin high mobility group protein 1 [23] and nylon beads [85]; however, the binding was not explicitly confirmed. Additionally, the clone KLWVIPQ was isolated by Wu *et al*. with the goal of selecting an NS2B (Dengue virus protease complex domain) mimetic, but this clone failed to function as such [98]. 

Because slightly diverse peptides as a part of common motif were usually selected, and because peptides with the same motif also emerged from the Ph.D.-12^TM^ library (Table 3), a propagation advantage of all corresponding phage clones is very unlikely.

In the majority of studies, the affinities of respective phagotopes toward their targets were confirmed. Additionally, BLAST searches identified numerous human and non-human proteins containing nearly exact matches to the heptapeptides [95,96,97]. Therefore, sequences corresponding to the K(L/V)WX(I/L/V)P motif could represent epitopes or “hot spots” common to various unrelated proteins, especially considering that W, P, Q and R are thought to be among the critical residues at protein-binding sites [99]. 

On the other hand, the high incidence of these sequences indicates they could be selection-related. It is possible that these clones are enriched on a target-unrelated common determinant due to poor target reactivity [6]. A likely suspect for a common determinant is the *E. coli* lipopolysaccharide (LPS) that remain in target preparations after their recombinant expression or in PEG-purified phage samples after amplification [100]. This assumption fits well with the recently described interaction between lipid A (a lipid component of LPS) and a designed LPS-neutralizing peptide. Analyses have revealed that peptide residues K3, L4, W5, F10, I11 and R12, which we find extremely similar to our motif, are engaged in interactions with lipid A [101]. If the target is the same in biopanning and affinity testing, lipopolysaccharide impurities could also be the cause for misinterpreted affinity. In future studies, the verification of K(L/V)WX(I/L/V)P binding to lipid A would be reasonable.

### 4.3. Peptide APWHLSSQYSRT

A phage clone displaying the peptide APWHLSSQYSRT was selected by various groups using phage display library the Ph.D.-12^TM^. Zahid *et al.* [102] demonstrated that this peptide is selective for targeting heart tissue. However, the same clone was also identified in biopanning on rhesus monkey embryonic [103] and derived neuronal stem cells [104]. In contrast to another peptide selected by the same group, binding of APWHLSSQYSRT was not cell specific [105]. The chance of the peptide having a common target on the mentioned cells is diminished by the fact that it was also selected in biopanning on apatite-based materials [105], sulfated glycoprotein HSO_3_-LeX [106], pancreatic phospholipase [93], ghrelin and β2-glycoprotein I (our work). Binding was confirmed only to apatite-based materials; however other selected peptides exhibited greater affinity. The authors confirmed that the peptide does not bind to polystyrene [105]. This peptide also emerged in selections that did not include polystyrene solid surfaces. Increased background binding can be observed when high titers of APWHLSSQYSRT-displaying phage are used [105]; however, this trend is common with a variety of phage clones. This peptide sequence does not resemble any other known target-unrelated motif. Analysis of selected clones by INFO (https://relic.bio.anl.gov/) denoted APWHLSSQYSRT as a low information content clone [105]. Information content is a statistical parameter that represents the possibility of observing a certain peptide by chance. Low information content peptides are more common in the phage library because they facilitate growth of phage clones and are likely to be selected due to propagation advantage. The selection of peptides with relatively high information content, which are less common in the library, is indicative for affinity-driven selection. This classification indicates that APWHLSSQYSRT could be a propagation-related target-unrelated peptide that emerged as a result of peptide composition or/and high growth rate.

### 4.4. Peptide HGWLYPHPRYPV

Another phage clone repeatedly selected from the Ph.D.-12^TM^ library in our laboratory displays the peptide HGWLYPHPRYPV. It was the most abundant clone selected on pancreatic phospholipase using specific elution with the reversible inhibitor MJ33 (1-Hexadecyl-3-(trifluoroethyl)-sn-glycero-2-phosphomethanol) [93]. Although binding to pancreatic phospholipase and related ammodytoxin C was confirmed with surface plasmon resonance, some doubts appeared when selections on β2-glycoprotein I and 17-β hydroxysteroid dehydrogenase also resulted in the enrichment of HGWLYPHPRYPV. The amino acid sequence does not match any known target-unrelated motif and did not appear in any other published scientific article.

### 4.5. Peptide LPLTPLP

A phage clone displaying the peptide LPLTPLP was isolated during our screening on β2-glycoprotein I and exhibited considerable affinity towards the target. However, we noticed that this particular heptapeptide emerged in several other experiments where the Ph.D.-7^TM^ library was used. 

This peptide was recovered in *in vivo* phage pannings aimed at identifying brain [86], heart [87,107] and skeletal muscle [87] homing peptides and was identified as a brain tissue specific binder. LPLTPLP was also selected in an *ex vivo* panning within human umbilical cords and exhibited moderate binding to fresh cord segments and human umbilical vein endothelial cells [73]. In a study by Lowe *et al*., the authors were aiming to identify peptides that bind or penetrate mouse zona pellucida. One of the *in vitro* selections performed on mouse embryos showed a definite bias (80%) towards this sequence [80]. 

The heptapeptide was further identified in screenings with a teratogenic serotonin receptor inverse agonist (SB-236057) [108], different rRNA targets [109] and the following protein targets: monoclonal anti-angiogenic antibody [110], melanoma inhibitory activity (MIA) protein [111], prion protein [112], chromatin high mobility group protein 1 [23] and an epitope on kidney-specific H-ATPase subunit G3 [113]. Although the binding of the LPLTPLP peptide to SB-236057, monoclonal anti-angiogenic antibody and MIA was not or could not be demonstrated [108,110,111], its affinity towards rRNA, prion protein and HMGB1 was confirmed by on-bead fluorescence assay, ELISA and Far-Western blotting, respectively [23,109,112]. Various authors have identified different proteins that contain regions with a high degree of homology to the selected LPLTPLP [108,112,113]. Accordingly, Rauch *et al.* identified r-esp1 nuclear factor and suggested that it has an essential role in the SB-236057 teratogenic mechanism [108]. Furthermore, based on LPLTPLP homology with the H-ATPase subunit a4 and confirmed binding of LPLTPLP to the epitope on H-ATPase subunit G3, Norgett *et al.* have suggested that the epitope directly interacts with and is therefore masked by the subunit a4 [113]. 

Because different screening systems used in the studies stated above lack a common component, it is very unlikely that LPLTPLP is a selection-related target-unrelated peptide. Its frequent occurrence could be partially explained by the amino acid biases of M13 libraries involving the over-representation of peptides containing P and T residues [8]. On the other hand LPLTPLP resembles to the P rich regions also known as “sticky arms”. The latter can bind rapidly and non-specifically to other proteins and therefore have an important role in protein interactions [114].

### 4.6. Peptide SVSVGMKPSPRP

Several authors have already suggested that the Ph.D.-12^TM^ SVSVGMKPSPRP peptide is a target-unrelated clone and more specifically, is a propagation-related target-unrelated peptide [115,116,117,118,119]. In correspondence with the Ph.D.-12^TM^ library provider, Kolb *et al.* have reported that the latter is probably due to the loss of the alpha-complementation segment present in phage DNA [115]. This notion is further supported by the fact that SVSVGMKPSPRP has been selected on many organic [80,120,121,122,123,124,125,126,127,128,129,130,131,132,133,134,135,136] and inorganic targets [119,137,138,139,140,141], yet its binding to the respective targets (Table 3) was not evaluated or could not be exclusively confirmed. 

However, it should also be noted that SVSVGMKPSPRP does exhibit affinity towards some targets and even performs its intended function. SVSVGMKPSPRP was identified as an HIV-1Vif- [142] and DNA-binding [143] peptide, as confirmed by ELISA. The peptide was also selected as a glucose oxidase binder, and the dissociation value was determined to be 0.0087 by SPR [144]. Shao *et al.* have isolated SVSVGMKPSPRP on liposomes prepared from phosphatidylserine and confirmed its selective binding to apoptotic cells by immunohistochemical staining [145]. The respective sequence was also shown to bind and was suggested to enter the cationic amino acid transporter-expressing cells [146]. In a study by Lin *et al.*, SVSVGMKPSPRP was identified as a mimotope of the conformational epitope on envelope (E) protein of the Japanese encephalitis virus (JEV). This identifiation was confirmed by competitive ELISA, plaque neutralization assay, and its ability to elicit JEV neutralizing antibodies in mice [147]. SVSVGMKPSPRP was also isolated in an *in vivo* phage display selection aimed to identify tumor blood vessels-targeting peptides. It was recognized that the peptide specifically targets tumor neovasculature endothelial cells and that the PSP motif is imperative for this targeting [148]. SVSVGMKPSPRP also exhibits binding affinity to the GaAs surface [149] and GaN surface [150] and has high specificity towards hydroxyapatite and tooth enamel [151].

SVSVGMKPSPRP is very likely a propagation-related target-unrelated peptides, considering the frequency of its incidence. However, we cannot exclude the possibility of SVSVGMKPSPRP being an actual binder for some targets, because quite a few motifs can be found within this dodecapeptide.

**Table 3:** Other frequently isolated peptides and motifs.

Selected peptide or consensus binding motif	Intended target and reference	Suspected decoy
	Bovine serum albumin [22]	
	Chromatin high mobility group protein 1 box A [23]	
	Chromatin high mobility group protein 1 box B [23]	
	Monoclonal antibody mAb 8C11 [77]	
HWGMWSY	Helix 9 of 16S rRNA of *Pseudomonas aeruginosa* [91]	Plastic/propagation related
	SPARC (osteonectin) [92]	
	Monoclonal antibody HmenB13 [152]	
	Ghrelin (our work)	
	Unacylated ghrelin (our work)	
	β2-glycoprotein I (our work)	
	Pancreatic phospholipase [93]	
HGWLYPHPRYPV	β2-glycoprotein I (our work)	Propagation related
	17-β hydroxysteroid dehydrogenase (our work)	
	Chromatin high mobility group protein 1 [23]	
	Nylon beads [85]	
	Pancreatic phospholipase [93]	
	IL-6 [94]	
	Mungbean’s heat shock proteins [95]	
K(L/V)WX(I/L/V)P	*E.coli* FtsA protein [96]	Lipid A
	N-terminal domain of human topoisomerase I [97]	
	Dengue viral protease [98]	
	β2-glycoprotein I (our work)	
	Lipoprotein lipase (our work)	
	Beta-ketoacyl-ACP reductase (our work)	
	Apatite based materials [86]	
	Pancreatic phospholipase [93]	
	H9C2 cardiomyoblast cell line [104]	
	Monkey embryonic stem cells [105]	
APWHLSSQYSRT	Neural stem cells derived from rhesus monkey embryonic stem cells [106]	Propagation related
	Sulphated glycoprotein HSO_3_-LeX [107]	
	β2-glycoprotein I (our work)	
	Ghrelin (our work)	
	Chromatin high mobility group protein 1 [23]	
	Human umbilical cords [73]	
	Mouse embryos [80]	
	Mouse brain tissue [86]	
	Mouse heart and skeletal muscle [87]	
	Rat heart [107]	
	Teratogenic serotonin receptor inverse agonist (SB-236057) [108]	
LPLTPLP	RNA [109]	Propagation related
	Monoclonal anti-angiogenic antibody [110]	
	Human melanoma inhibitory activity (MIA) protein [111]	
	Prion protein [112]	
	Peptide of kidney-specific H-ATPase subunit G3 [113]	
	β2-glycoprotein I (our work)	
	Mouse ova and embryos [80]	
	InP [119]	
	Hair and skin [120]	
	Bevacizumab-treated tumors [121]	
SVSVGMKPSPRP	Cultured mouse cerebellar granule neurons [122]	Propagation related
	Human prostate cancer cells (DU145) [123]	
	HepG2 (liver cells) [124]	
	GlyR-expressing cells [125]	
	*Staphylococcus aureus* [126]	
	Peptides that bind to Hsp 70-associated antigens [127]	
	HIV directed monoclonal antibody 2G12 [128]	
	Anti-EG95 antibodies [129]	
	Human neonatal IgM antibodies [130]	
	Nerve growth factor [131]	
	Acetylcholinesterase [132]	
	Trans-activation responsive element RNA [133]	
	Alzheimer's disease amyloid peptide Abeta(1-42) [134]	
	Murine monoclonal antibody, 9-2-L379 specific for meningococcal lipo-oligosaccharide [135]	
	Cerebrospinal fluid antibody [136]	
SVSVGMKPSPRP	Single-walled carbon nanotube [137]	Propagation related
	FePt [138]	
	Cobalt nanoparticles [139]	
	Polytetrafluoroethylene [140]	
	Ink pigments [141]	
	HIV-1 Vif [142]	
	DNA [143]	
	Glucose oxidase [144]	
	Liposomes prepared from phosphatidylserine [145]	
	Cationic amino acid transporter expressing cells [146]	
	Japanese encephalitis virus neutralizing antibody [147]	
	Mouse tumor blood vessels [148]	
	GaAs surface [149]	
	GaN surface [150]	
	Hydroxyapatite [151]	

## 5. How To Recognize and Avoid Target-Unrelated Peptides

Although a screening system always incorporates several components (other than the target molecule) and some phage display libraries evidently contain phage clones with propagation advantages, in the majority of selections, target unrelated peptides do not appear or at least do not predominate. Experimental conditions affect the selection process, and careful design of experiments can reduce the possibility of selecting target-unrelated peptides. 

Binding to plastic can be reduced, to some extent, by blocking the surface or by using alternative methods for target immobilization instead of direct plastic adsorption. Immobilization of a target at a higher density also decreases the possibility for selecting background binders [9]. However, higher target density will reduce the stringency of screening [3], which may preferentially lead to the selection of more abundant low or moderate affinity binders. Another strategy to remove background binders is subtractive selection. The input phage pool is incubated with a capturing and blocking agent coated on the solid phase (subtractive selection) prior to the target in all selection rounds [153]. Yet another approach to eliminate target-unrelated peptides is specific elution with known target ligands and should be applied if possible [3]. 

Phage clones with propagation advantages are outnumbered in the library by clones with normal propagation rates and are therefore likely to be eliminated by a population bottleneck created in affinity-driven selections [5]. In the case of poor affinity selections, no specific binders are enriched and no population bottleneck is present. Under such conditions even rare propagation-related target-unrelated peptides are given an opportunity to predominate, especially if many amplification steps are performed. Therefore serial amplification of a library should be avoided. Amplification of the input phage after negative selection step in subtractive biopanning is also not recommended. In libraries based on fd-tet inspection for larger and slightly less turbid plaques enables approximate monitoring for presence of fast propagating clones [5]. Another option is to use T7 lytic phage-displayed peptide libraries, which exhibit less sequence bias in comparison to M13 phage-displayed peptide libraries [8]. T7 phage particles are released from host cells by lysis so membrane transport cannot affect phage particles assembly. Displayed peptides are less likely to interfere with the phage replication process, which also lessens the possibility for the existence of radically faster propagating phage clones.

Despite all the precautions it is impossible to completely avoid selection of target-unrelated peptides. Therefore, discrimination between these peptides and specific binders is very important. Background binders may especially contribute falsely to apparent affinity toward the target and give misleading results because the biopanning and binding assay (usually ELISA) often employs the same compounds [7]. The obtained results can therefore not be fully trusted. Other methods, such as surface plasmon resonance (SPR) or *in vivo* experiments can eliminate unrelated peptides; however, these methods are less appropriate for rapid screening of large number of phage clones. Separately testing selected clones for binding to all components present during the screening separately is recommended. 

Recently, a web server named SAROTUP, an acronym for “Scanner And Reporter Of Target-Unrelated Peptides,” has been established [154]. Although it was developed for cleaning the output data of phage display selections on antibodies, it is of course a useful tool for selections on any other screening target. SAROTUP is freely accessible (http://immunet.cn/sarotup/) and capable of finding, reporting, and excluding possible target-unrelated peptides. When using SAROTUP for cleaning the data, the user uploads a set of peptide sequences in FASTA format. The tool compares each sequence with its database, and potential target-unrelated peptides are reported in a table. Although SAROTUP represents a time-efficient, cost-free and user-friendly approach for the evaluation and cleaning of results, it must be used with caution. False negative and false positive outcomes are still possible. For example, the His-enriched clone HHHHPT (Ni^2+^ binding peptide) was not recognized as a target-unrelated sequence. Moreover, the SAROTUP database is rather limited; it recognizes only 23 target-unrelated motifs. Expanding the database by adding new motifs is essential to further improve the usefulness and accuracy of this tool. Another approach for identification of target-unrelated peptides is searching databases for peptides selected on different targets. MimoDB [116] and PepBank [118] are freely accessible databases that contain large numbers of peptides obtained with phage display. Distinguishing target-unrelated peptides from true binders is also possible by calculating the information measure of each peptide [155] using the INFO program (https://relic.bio.anl.gov/), which represents the possibility of observing a certain peptide by chance. Phage clones with a propagation advantage display peptides with lower information content. 

Distinguishing true binders from false positives is an important step toward greater integrity of phage display selection. To achieve this, selected peptides should be carefully examined, compared to known target-unrelated motifs, tested for background binding and assessed for possible propagation advantages. This review provides a list of already recognized target-unrelated peptides, discusses ambiguous peptides or peptides motifs, suspected of being nonspecific for the target and suggests strategies to avoid isolation of such peptides. Thus certain web tools also enable examination and comparison of selected peptides to known target-unrelated motifs.

## 6. Conclusions

Even with the technology as successful as phage display, which generated many strong ligands for various targets, false positive results occur. These usually emerge during selections with targets for which only low/moderate affinity ligands exist in a phage display library, and are either selection or propagation related. Distinguishing true binders from false positives is an important step toward greater integrity of phage display selection. To achieve this, selected peptides should be carefully examined, compared to known target-unrelated motifs, tested for background binding and assessed for possible propagation advantages. This review provides a list of already recognized target-unrelated peptides, discusses ambiguous peptides or peptides motifs, suspected of being nonspecific for the target and suggests strategies to avoid isolation of such peptides.
